# Treatment with Fluticasone Propionate Increases Antibiotic Efficacy during Treatment of Late-Stage Primary Pneumonic Plague

**DOI:** 10.1128/AAC.01275-21

**Published:** 2022-01-18

**Authors:** Samantha D. Crane, Srijon K. Banerjee, Roger D. Pechous

**Affiliations:** a Department of Microbiology and Immunology, University of Arkansas for Medical Sciences, Little Rock, Arkansas, USA

**Keywords:** *Yersinia pestis*, corticosteroids, fluticasone propionate, inflammation, neutrophils, plague, pneumonia, pneumonic plague

## Abstract

Severe and late-stage pneumonias are often difficult to treat with antibiotics alone due to overwhelming host inflammatory responses mounted to clear infection. These host responses contribute to pulmonary damage leading to acute lung injury, acute respiratory distress syndrome, and death. In order to effectively treat severe and late-stage pneumonias, use of adjunctive therapies must be considered to reduce pulmonary damage when antimicrobial agents can be administered. Pneumonic plague, a severe pneumonia caused by inhalation of Yersinia pestis, is a fatal disease that causes death within 6 days without antibiotic intervention. Late-stage pneumonic plague is difficult to treat, as antibiotics must be delivered within 24 h after onset of symptoms to be effective. Here, we use a murine model of primary pneumonic plague to examine how host inflammatory responses impact antibiotic treatment of late-stage pneumonic plague. We developed a murine infection model demonstrating the poor outcomes associated with delayed delivery of antibiotics. We show that pretreatment of mice with intranasal fluticasone propionate increased the efficacy of delayed antibiotic delivery and enhanced murine survival. Mice receiving fluticasone propionate also showed decreased bacterial burden and reduced inflammatory pathology in the lungs. Further, we show that treatment and survival correlated with decreased levels of interleukin-6 (IL-6) and reduced neutrophil infiltration to the lungs. This work demonstrates how host inflammatory responses complicate treatment of late-stage pneumonic plague and suggests that targeting of host inflammatory responses may improve treatment of severe, late-stage pneumonia.

## INTRODUCTION

Pulmonary infections cause significant disease burdens worldwide and are reported to reduce disability-adjusted years of life lost more than cancer, diabetes, and many other infectious diseases (1). Pneumonia causes a massive burden on health care systems, costing greater than $9.5 billion in 2013 in the United States (2), and has garnered much attention recently as a complication in individuals with COVID-19 (3). Pneumonia resulting from pulmonary infection that causes inflammation and fluid accumulation in lungs can severely impact respiratory function, sometimes causing acute lung injury (ALI), acute respiratory distress syndrome (ARDS), and death (1, 4–6). As a result, severe, late-stage pneumonias can often be difficult to treat with antimicrobial agents alone due to the dramatic and ultimately damaging host responses mounted to clear microbial infections.

While many microbes cause pneumonia, the disease is largely defined by the host response to infection. The host response begins with pathogen recognition by alveolar macrophages and other cells residing in the alveolar space. Upon recognition of a pathogen, cell signaling events occur that induce expression of cytokines and chemokines, such as tumor necrosis factor alpha (TNF-α), interleukin-6 (IL-6), IL-8, and CXCL5, which prime the alveolar space and recruit critical innate immune populations to the airways (1). Neutrophils are one of the earliest-responding cells and are recruited to eliminate microbial insults by processes such as phagocytosis and release of antimicrobial mediators by degranulation. While critical to combating infection, neutrophils can also promote development of severe pneumonia by release of toxic antimicrobial agents, including reactive oxygen species (ROS), proteinases, and cationic polypeptides (7, 69). Innate immune populations are typically able to control and clear infection, and a resolving phase is initiated that includes efferocytosis of neutrophils and repair of pulmonary damage. If unresolved, the inflammatory host-derived agents can promote pulmonary damage and lead to ALI, ARDS (8), and death.

Pneumonic plague is caused by pulmonary infection with the bacterium Yersinia pestis and is 100% lethal within a week after exposure without antibiotic intervention (9). Further, antibiotic treatment must be administered within 24 h after the onset of symptoms to be effective (10). Pneumonic plague initially manifests with nonspecific flu-like symptoms within this time frame, making appropriate diagnosis and timely treatment difficult. Infected individuals ultimately succumb to a deadly pneumonia caused by a massive influx of immune cells and subsequent pulmonary tissue damage. We predicted that the rapid onset of inflammatory responses complicate treatment of late-stage pneumonic plague (after the onset of symptoms) by compromising pulmonary function before antibiotics are able to control infection. In this study, we investigate how broadly suppressing host inflammatory responses impacts the treatment of pneumonic plague. We show that pretreatment with fluticasone propionate (Fp) in combination with delayed (and otherwise ineffective) antibiotic administration results in increased survival in a murine infection model of pneumonic plague compared to antibiotic alone. We show that Fp influences host innate immune responses to Y. pestis pulmonary infection by limiting neutrophil infiltration into the airways and reducing lung concentrations of IL-6. This work shows that host inflammatory responses contribute to difficulty treating late-stage pneumonic plague and suggests that immunomodulatory therapies coupled with antibiotic delivery may increase treatment efficacy.

## RESULTS

### Neutrophils and treatment of pneumonic plague.

**FIG 1:**
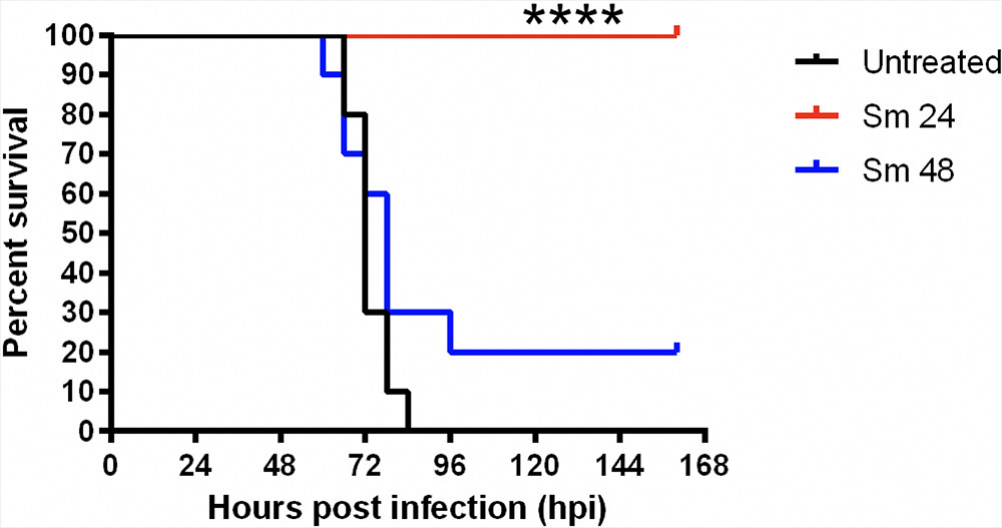
Delayed treatment of pneumonic plague decreases murine survival. C57BL/6 female mice (*n* = 10 to 14 per group) were infected with 10^4^ CFU of wild-type CO92 Y. pestis via the intranasal route. Mice were either left untreated or treated via the intraperitoneal route with streptomycin (Sm) beginning at 24 or 48 hpi and then every 8 h thereafter. Mouse survival over a period of 160 h was recorded. Significance was calculated using a log rank test: ****, *P* ≤ 0.0001. The data presented were pooled from two experiments that are representative of multiple repeats.

Similar to pneumonia caused by other microbes, successful treatment of pneumonic plague becomes increasingly difficult the longer antibiotic administration is delayed after symptoms present. In humans, it is recommended to begin antibiotic treatment within the first 24 h after the presentation of symptoms to survive disease (10–12). We hypothesized that difficulty treating pneumonic plague after the onset of symptoms may be attributed to pulmonary damage caused by overzealous immune responses. To examine this, we established a murine infection and treatment model that mimics clinical treatment of late-stage pneumonic plague. Female 4- to 6-week-old C57BL/6 mice were inoculated with a lethal dose of 10^4^ CFU of fully virulent Y. pestis strain Colorado 92 (CO92) via the intranasal (i.n.) route and either left untreated or were treated with 160 mg/kg of body weight streptomycin (Sm) beginning at 24 h postinfection (hpi) (early [Sm 24 group]) or 48 hpi (late [Sm 48 group]). Streptomycin has consistently demonstrated efficacy in treatment of plague (13, 14). Whereas previous models have demonstrated success of initiating streptomycin therapy at 42 hpi (13), we sought to model initiation of therapy later, when efficacy was severely diminished. As expected, we observed death of all infected untreated mice by 72 hpi and survival of all mice receiving streptomycin beginning at 24 hpi (Fig. 1). Twenty percent of mice receiving Sm treatment at 48 hpi survived infection; however, this survival was not significantly different from survival of untreated mice, effectively modeling the decreased success of delayed antibiotic treatment. Though a representative of multiple repeated experiments is shown in Fig. 1, we saw anywhere between 0 and 20% survival for treated mice receiving antibiotics beginning at 48 hpi.

**FIG 2:**
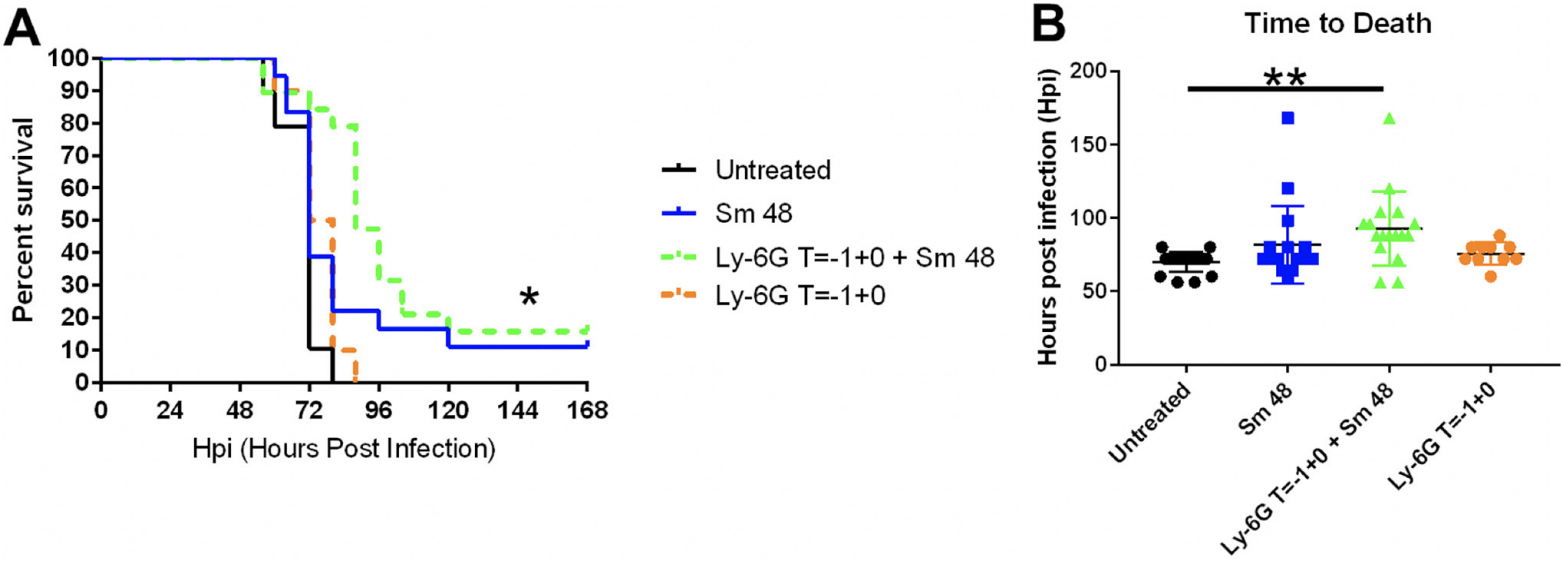
Neutrophils complicate treatment of pneumonic plague. (A) C57BL/6 female mice (*n* = 19 to 20 per group) were infected with 10^4^ CFU of wild-type CO92 Y. pestis via the intranasal route. Mice were either left untreated, treated with streptomycin (Sm) beginning at 48 hpi, given anti-Ly-6G 1 day prior to and at the time of infection, or given anti-Ly-6G 1 day prior to and at the time of infection in conjunction with Sm treatment at 48 hpi. Mouse survival over a period of 7 days was recorded. Surviving mice at 7 days postinfection (dpi) were sacrificed. Significance was calculated using the log rank test: *, *P* ≤ 0.05. (B) Time to death was determined using survival data from panel A, with mice surviving at 7 dpi included and with time to death recorded as 168 hpi. Significance was calculated using one-way ANOVA: **, *P* ≤ 0.005. Error bars represent standard deviation (SD). The data presented were pooled from two independent experiments.

To test if host responses complicate treatment of late-stage infection, we sought to limit and/or suppress host inflammatory responses to see if we could improve survival outcomes after delayed antibiotic treatment. Previous work showed that neutrophil accumulation is largely responsible for formation of inflammatory lesions that grow to encompass entire lobes of the lung and ultimately compromise pulmonary function (15). We therefore tested whether depletion of neutrophils enhanced treatment of late-stage pneumonic plague. We treated two groups of mice with anti-mouse Ly-6G (anti-Ly-6G) antibody 1 day prior to and on the day of infection to deplete neutrophils (see Fig. S1 in the supplemental material). Antibiotic treatment was then initiated at 48 hpi for both groups. Finally, additional groups of mice were infected and left untreated or treated with antibiotics beginning at 48 hpi for infection control and standard treatment comparisons, respectively. Mice receiving anti-Ly-6G antibody all succumbed to disease at a similar time as untreated mice, indicating that neutrophil-depleting antibody alone does not significantly improve survival of pneumonic plague (Fig. 2A). Mice receiving anti-Ly-6G in conjunction with Sm at 48 hpi exhibited significantly increased survival compared to untreated mice, as well as an increased mean time to death (Fig. 2A and B). Pretreatment with anti-Ly-6G in conjunction with Sm at 48 hpi, though, did not significantly impact either parameter compared to antibiotic alone. Together, these data show that abatement of early neutrophil influx combined with delayed antibiotic treatment did not significantly improve treatment with antibiotic alone, and therefore neutrophils are likely not responsible for difficulty treating late-stage infection.

### Adjunctive Fp pretreatment and late Sm treatment increase murine survival of pneumonic plague.

**FIG 3:**
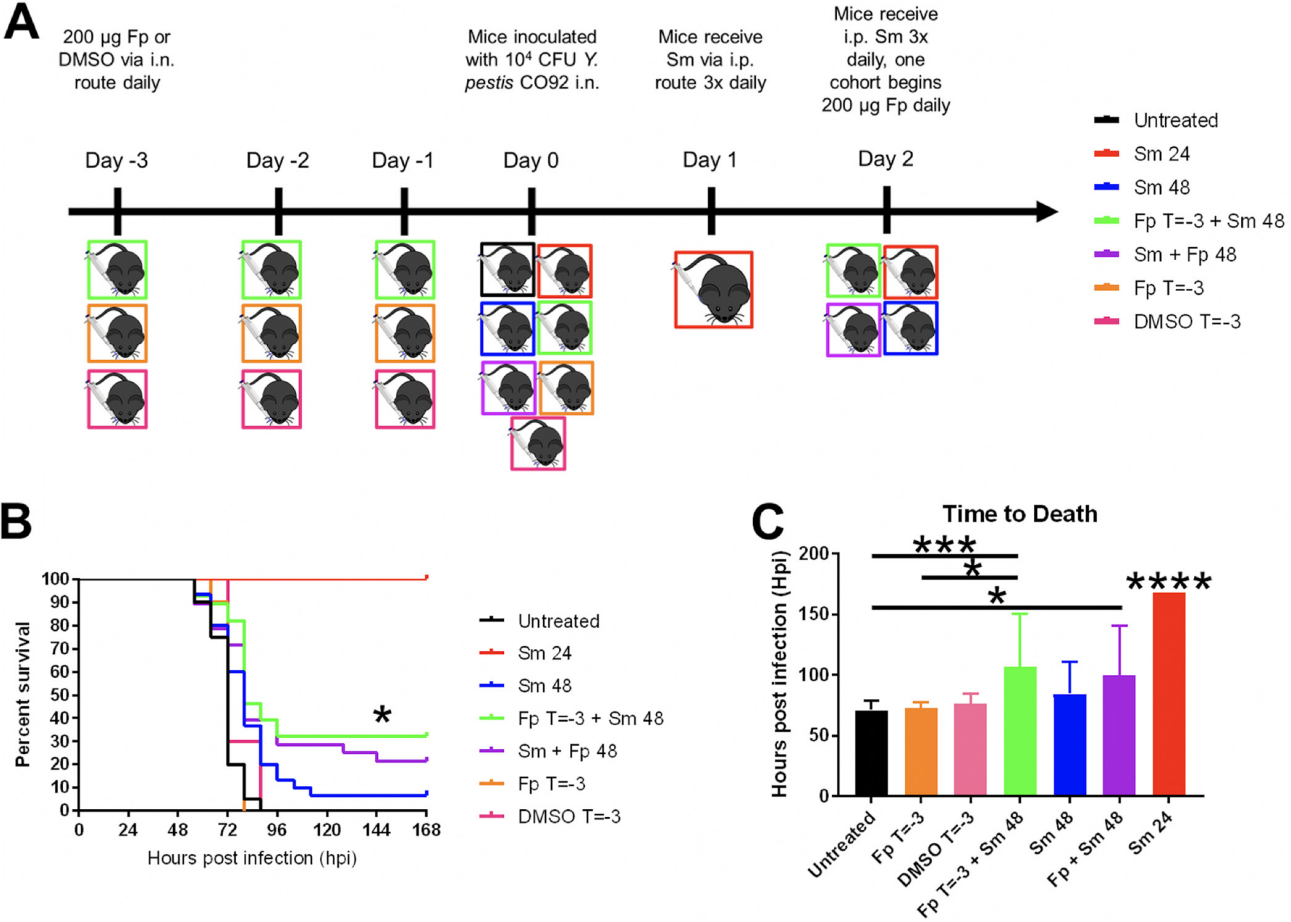
Adjunctive Fp pretreatment and late Sm treatment increase murine survival of pneumonic plague. (A) Treatment regimen of mice receiving intranasal (i.n.) Fp and intraperitoneal (i.p.) Sm treatment prior to and during Y. pestis infection. Mice were infected with 10^4^ CFU of Y. pestis and were left untreated, received streptomycin (Sm) beginning at the indicated time (and every 8 h after), fluticasone propionate prior to and daily during infection (Fp T = −3 + Sm 48 h), fluticasone propionate at the same time as streptomycin (Fp + Sm 48 h), fluticasone propionate only, or DMSO. (B) Survival was monitored over the course of 7 days. Surviving mice were sacrificed at 7 dpi. Significance was calculated with the log rank test: *, *P* < 0.05. (C) Time to death was calculated using survival data from panel B, with mice surviving at 7 dpi included and with time to death recorded as 168 hpi. Significance was calculated by one-way ANOVA: *, *P* ≤ 0.05; **, *P* ≤ 0.005; ***, *P* ≤ 0.0005; ****, *P* ≤ 0.0001. Error bars represent SD. The data presented were pooled from three independent experiments.

The finding that early neutrophil influx did not significantly improve treatment led us to investigate whether a more global approach to immunosuppression could better enhance survival. To this end, we used a commercially available corticosteroid, fluticasone propionate (Fp), as a treatment to globally dampen immune responses. Fp is a trifluorinated glucocorticoid with anti-inflammatory effects in allergen responses as well as pulmonary Klebsiella pneumoniae and Mycoplasma pneumoniae infection models (16–18). Seven cohorts of C57BL/6 mice were used to determine whether Fp treatment during pneumonic plague could increase murine survival (Fig. 3A). In addition to the untreated, Sm 24, and Sm 48 cohorts shown in Fig. 1, groups of mice were pretreated via intranasal inoculation with Fp or vehicle (dimethyl sulfoxide [DMSO]) once every 24 h beginning 3 days prior to (T = −3 group) and following intranasal inoculation with Y. pestis. One of the cohorts receiving Fp then received Sm treatment beginning at 48 hpi (Fp T = −3 + Sm 48 group), while the other received Fp treatment only (Fp T = −3 group). These groups were designed to determine whether the absence of host inflammatory responses improved the effectiveness of antibiotics delivered late during infection. We also investigated the delivery of Fp and Sm (Fp + Sm 48) simultaneously in one group of mice to determine if corticosteroid/antibiotic cotreatment might serve as a clinically relevant treatment option. As expected, we observed death of all untreated mice before 96 hpi (Fig. 3B), and mice receiving Sm at 24 hpi all survived to the end of the time course. The DMSO (Fp vehicle) T = −3 and Fp T = −3 treatment groups also showed similar survival compared to untreated mice, showing that intranasal treatment with vehicle or Fp alone did not improve survival outcomes. While some mice in the Sm 48 and Fp + Sm 48 groups did survive infection, overall survival was not significantly increased compared to untreated mice. In contrast, the Fp T = −3 + Sm 48 cohort showed significant survival compared to both untreated and Sm 48 (antibiotic alone) cohorts. We also observed (Fig. 3C) increased time to death in Fp T = −3 + Sm 48-treated mice compared to untreated and Fp T = −3 cohorts. Cotreatment with Fp and Sm initiated simultaneously also significantly increased time to death compared to that of untreated mice, indicating that both pretreatment and cotreatment with fluticasone enhanced survival. These data show that Fp pretreatment coupled with delayed Sm administration increased murine survival of pneumonic plague and enhanced survival compared to untreated mice and mice treated with antibiotic alone. These results indicate that host inflammatory responses complicate antibiotic efficacy to treat late-stage pneumonic plague.

### Fp and Sm treatments decrease lung Y. pestis burden.

**FIG 4:**
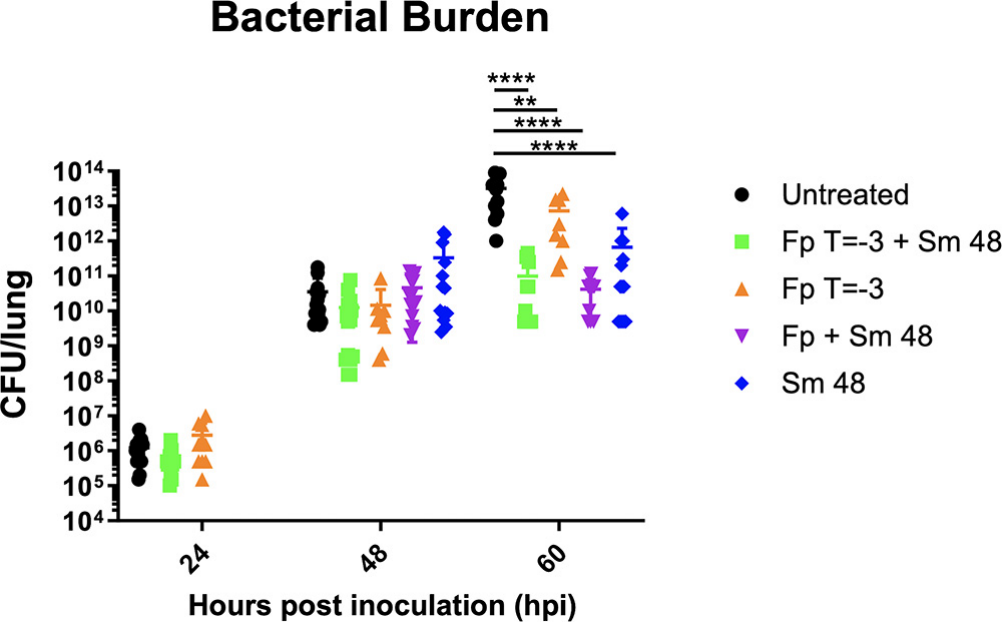
Fp and Sm treatment decrease lung Y. pestis burden. C57BL/6 female mice (*n* = 10 to 15 per group per time point) were infected with 1 × 10^4^ CFU of wild-type CO92 Y. pestis via the intranasal route and treated with the indicated regimens. At indicated time points postinfection, bacterial burdens were determined by lung homogenization plating of serial dilutions of lung homogenate. Error bars represent SD. Significance was calculated by two-way ANOVA: *, *P* ≤ 0.05; **, *P* ≤ 0.005; ****, *P* ≤ 0.0001. The data presented were pooled from three independent experiments.

We next sought to determine the impacts of the various treatments on bacterial survival during infection. We evaluated bacterial burdens in lungs from untreated, Fp T = −3 + Sm 48, and Fp T = −3 cohorts at 24 hpi and in untreated, Fp T = −3 + Sm 48, Fp T = −3, Fp + Sm 48, and Sm 48 cohorts at 48 and 60 hpi. At 24 or 48 hpi, we did not observe any change in bacterial burden in any cohort observed (Fig. 4). We did, however, observe differences in bacterial burden at 60 hpi in all groups receiving Sm at 48 hpi (Fp T = −3 + Sm 48, Fp + Sm 48, and Sm 48) and the Fp T = −3 cohort. Groups receiving Sm and either pretreatment or cotreatment with Fp showed the lowest bacterial burdens, though these values were not statistically significantly different from Sm alone. Bacterial burden in the Fp T = −3 cohort was significantly lower than burdens in untreated mice, showing that Fp pretreatment alone affects bacterial survival in murine lungs at 60 hpi. Together, these data show that Fp pretreatment and Sm treatment beginning at 48 hpi significantly reduce Y. pestis lung burdens at 60 hpi, indicating that treatment with Fp in addition to Sm may have the greatest impact on bacterial survival in the lung.

### Fp pretreatment impacts cytokine and chemokine profiles in lung.

**FIG 5:**
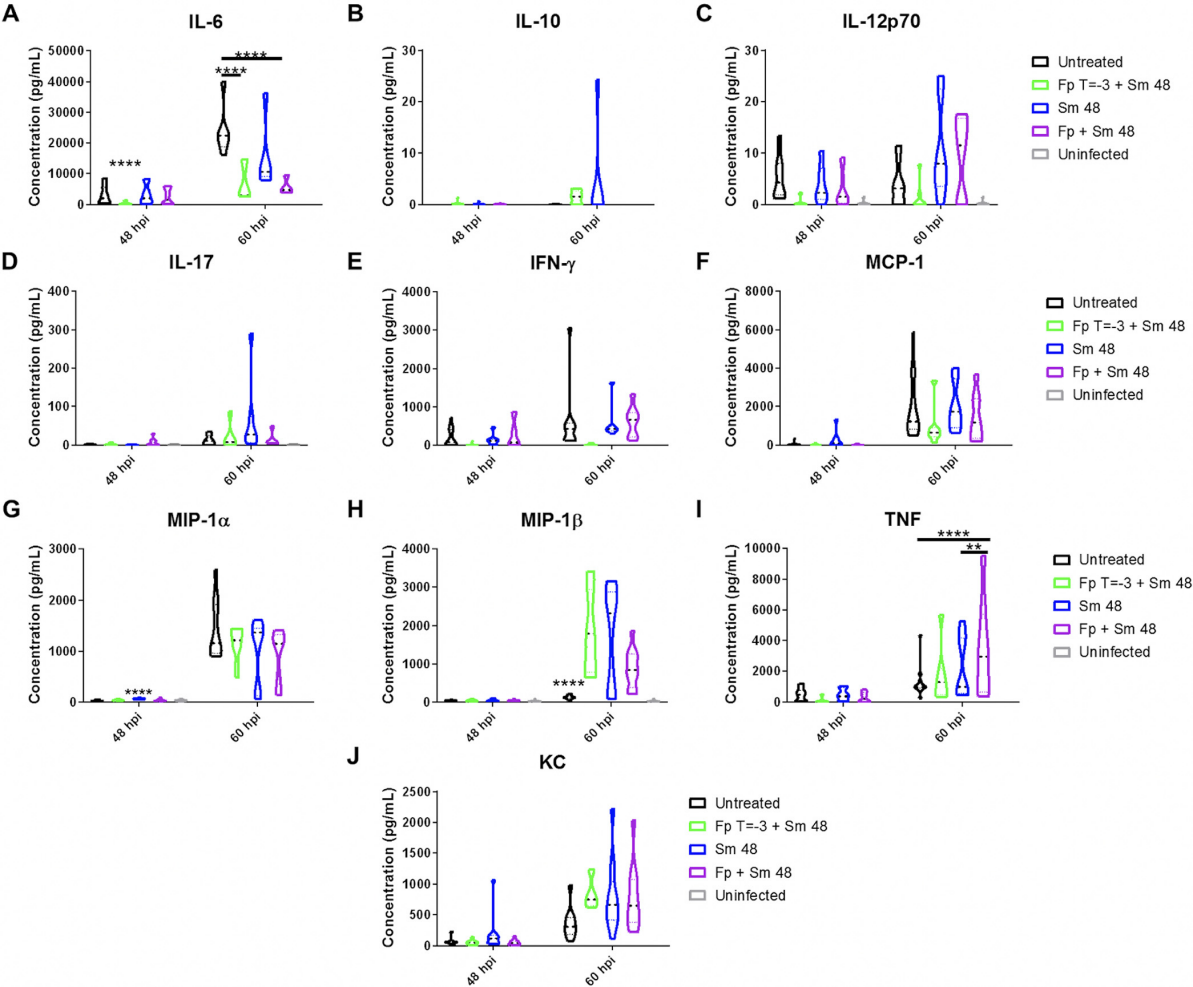
Fp pretreatment impacts cytokine and chemokine profiles in lung. C57BL/6 female mice (*n* = 9 to 15 per group per time point) were infected with 5 × 10^4^ CFU of wild-type CO92 Y. pestis via the intranasal route and treated with the indicated regimens. After the indicated times postinfection, mice were sacrificed and bronchoalveolar lavage fluid (BALF) was obtained from lungs. BALF samples were spun to obtain supernatants containing cytokines and chemokines. Cytokine bead arrays were performed to determine concentrations of (A) IL-6, (B) IL-10, (C) IL-12p70, (D) IL-17, (E) IFN-γ, (F) MCP-1, (I) TNF, and (J) KC. ELISAs were performed to determine concentrations of (G) MIP-1α and (H) MIP-1β. Error bars represent SD. ND, not detected. Significance was calculated by two-way ANOVA and *post hoc* Tukey’s test: *, *P* ≤ 0.05; **, *P* ≤ 0.005; ***, *P* ≤ 0.0005; ****, *P* ≤ 0.0001. The data presented were pooled from three independent experiments.

Increased survival in the Fp T = −3 + Sm 48 treatment group afforded us the opportunity to compare this and other cohorts to identify host responses that correlate with survival during treatment of pneumonic plague. We assayed concentrations of known proinflammatory chemokine and cytokines, including IL-6, IL-10, IL-12, IL-17, gamma interferon (IFN-γ), monocyte chemoattractant protein 1 (MCP-1), macrophage inflammatory protein 1α (MIP-1α), MIP-1β, TNF, and keratinocyte chemoattractant (KC) (Fig. 5A to J) in bronchoalveolar lavage fluid (BALF) obtained from mice at 48 and 60 hpi. Several of these molecules have previously been shown to be upregulated during the proinflammatory phase of pneumonic plague (19–22). Of all of these cytokines/chemokines, we only saw a consistent difference between the Fp T = −3 + Sm 48 and untreated groups for the cytokine IL-6. We observed significantly lower concentrations of IL-6 in the Fp T = −3 + Sm 48 cohort compared to all other cohorts at 48 hpi and significantly decreased IL-6 levels in Fp pre- and cotreatment groups compared to untreated mice at 60 hpi (Fig. 5A). We observed a higher concentration of MIP-1α in Fp T = −3 + Sm 48 animals compared to uninfected controls at 24 hpi (data not shown) and higher concentrations of MIP-1α in Sm 48 mice compared to all other groups at 48 hpi, though this difference was not apparent at 60 hpi. Together, our data show that Fp pretreatment with delayed Sm administration reduces BALF IL-6 concentrations. These results suggest that despite the ability of fluticasone to broadly suppress host inflammatory responses, reduced IL-6 specifically correlated with increased survival during infection and treatment.

### Impact of IL-6 knockout on treatment of pneumonic plague.

**FIG 6:**
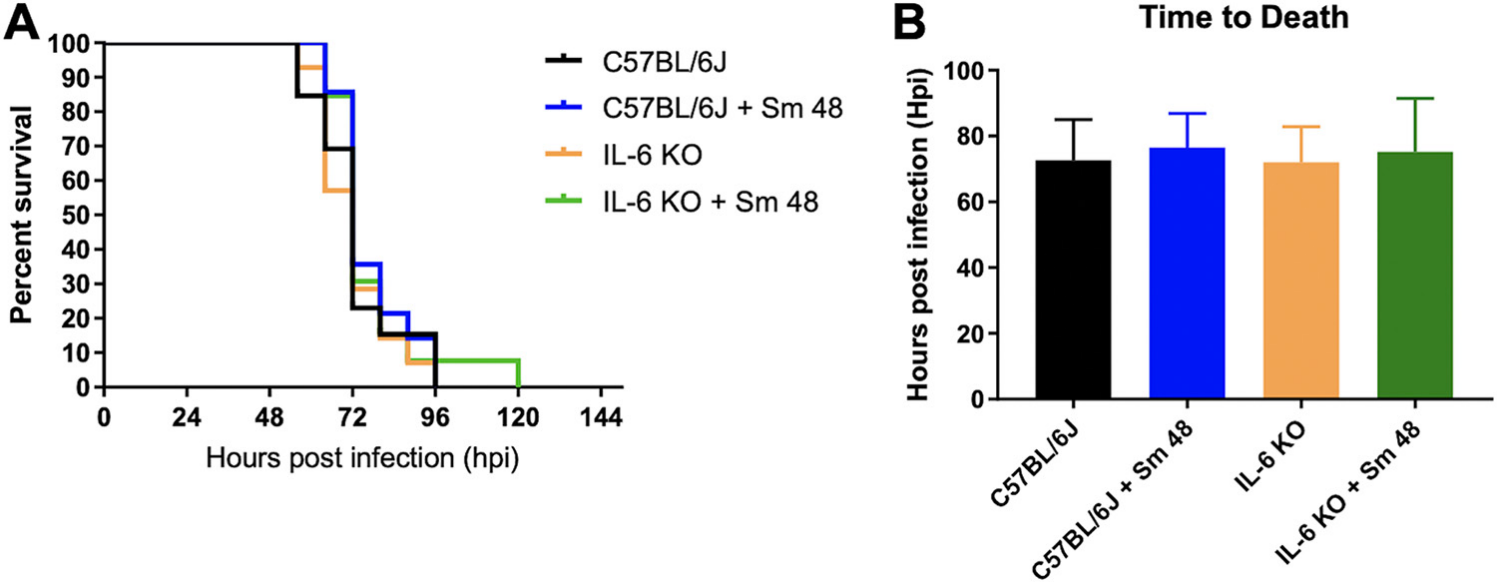
Absence of IL-6 does not affect treatment of pneumonic plague. C57BL/6J and IL-6 knockout mice (*n* = 14 to 15 per group) were infected with 10^4^ CFU of Y. pestis CO92 and treated with the indicated regimen. (A) Survival was monitored over time, and (B) time to death was determined. The data presented were pooled from two independent experiments.

The finding that lower BALF IL-6 concentrations correlated with increased survival of Fp T = −3 + Sm 48-treated mice led us to question whether IL-6 drives inflammation associated with lethality in the event of delayed treatment of pneumonic plague. To investigate this, we infected wild-type (C57BL/6J) and IL-6 knockout (KO) mice with 10^4^ CFU of CO92 Y. pestis via the intranasal route and left them untreated or administered delayed Sm treatment at 48 hpi (Sm 48) and observed survival over time. The absence of IL-6 had no significant effect on murine survival of pneumonic plague (Fig. 6A) regardless of treatment regimen. The mean times to death of these cohorts were also unaffected (Fig. 6B). These data suggest that IL-6-mediated inflammatory responses alone are not responsible for the difficulty treating late-stage pneumonic plague.

### Fp pretreatment impacts neutrophil recruitment to the lung.

We also sought to determine differences in pulmonary cell populations between untreated, Fp T = −3 + Sm 48, Sm 48, and cotreatment cohorts to determine if the presence of specific cell types in the lung correlated with increased survival and/or increased lethality. To this end, we assayed the frequency of alveolar macrophages, interstitial macrophages, CD11b^+^ CD11c^+^ macrophages, neutrophils, CD3^+^ cells, dendritic cells, and inflammatory monocytes at 48 and 60 hpi in the BALF of these groups of mice (see Fig. S2 in the supplemental material).

**FIG 7:**
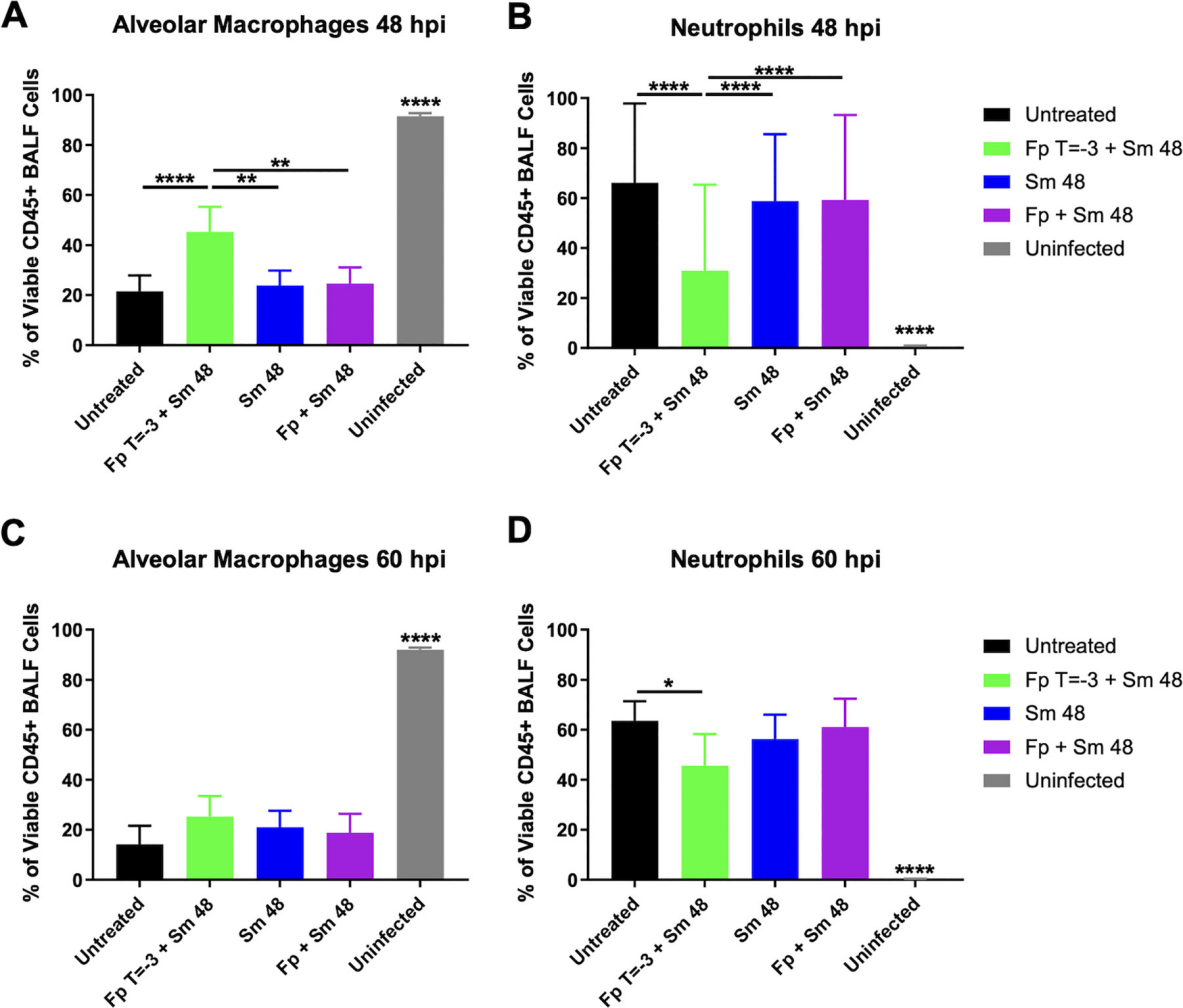
Fp pretreatment impacts neutrophil recruitment to the lung. C57BL/6 female mice from Fig. 3 (*n* = 9 to 15 per group per time point) were infected with 5 × 10^4^ CFU of wild-type CO92 Y. pestis via the intranasal route and treated with the indicated regimens. Proportions of alveolar macrophages (F4/80^+^ CD11b^mid/low^ CD11c^high^) and neutrophils (CD45^+^ F4/80^−^ CD11c^low^ CD11b^high^ Ly-6G^+^) in the BALF of each mouse were determined by flow cytometry at (A) 48 hpi and (B) 60 hpi. Error bars represent SD. Significance was calculated by two-way ANOVA: *, *P* ≤ 0.05; **, *P* ≤ 0.005; ****, *P* ≤ 0.0001. The data presented were pooled from three independent experiments.

At 48 hpi, we saw changes in the percentages of alveolar macrophages and neutrophils in all infected mice (Fig. 7A and B). While the BALF is largely comprised of alveolar macrophages in uninfected mice, the percentage of BALF cells that were alveolar macrophages was significantly lower for all groups of infected mice due to increased neutrophil infiltration in response to infection. The BALF of the Fp T = −3 + Sm 48 cohort had a significantly larger percentage of alveolar macrophages (Fig. 7A and B) and a corresponding decreased level of neutrophils compared to other infected groups. At 60 hpi, we observed decreased alveolar macrophage frequency in all cohorts compared to uninfected mice (Fig. 7C), which was again reflected in an increased percentage of BALF cells that were neutrophils (Fig. 7D). Despite this, we observed a significantly decreased frequency of neutrophils in the Fp T = −3 + Sm 48 cohort compared to untreated mice. Together, these data show that Fp administration resulted in decreased neutrophil infiltration into the alveolar spaces during the later stages of infection. These results suggest that reduced neutrophil infiltration correlates with increased survival during treatment of pneumonic plague.

### Y. pestis pulmonary infection impacts lung function parameters.

**FIG 8:**
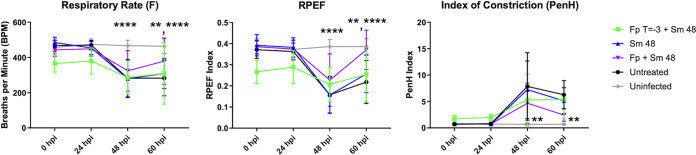
Y. pestis pulmonary infection impacts lung function parameters. C57BL/6 female mice (*n* = 10 to 12 per group per time point) were infected with 1 × 10^4^ CFU of wild-type CO92 Y. pestis via the intranasal route and treated with the indicated regimens. Whole-body plethysmography readings were taken at the indicated time points for 10 min to determine flow at 50% expired breath volume (EF50), RPEF, and index of constriction (PenH). Error bars represent SD. Significance was calculated by two-way ANOVA: **, *P* ≤ 0.005; ****, *P* ≤ 0.0001. The data presented were pooled from three independent experiments.

We next sought to determine how the treatment regimens impacted pulmonary function by using whole-body plethysmography. Whole-body plethysmography is a noninvasive approach that measures how inspiration and expiration cycles modify pressure in a specialized chamber. This method has been used extensively to characterize murine lung function in a variety of viral infection models (23–28). We measured several respiratory function parameters, including respiratory rate (F), RPEF (ratio of time to peak expiratory flow relative to total expiratory time), and the index of constriction (PenH) in untreated, Fp T = −3 + Sm 48, Sm 48, and Fp + Sm 48 cohorts at 24, 48, and 60 hpi (23). That PenH, an indirect measure suggestive of airway resistance, was increased, combined with a decrease in RPEF, suggests increased airflow resistance, as seen during pulmonary infection with severe acute respiratory syndrome coronavirus (SARS-CoV) and influenza virus A (23, 29), and intranasal inoculation with Y. pestis dramatically impacted most of the parameters of pulmonary function we tested for all infected groups, including respiratory rate, RPEF, and PenH compared to uninfected mice (Fig. 8). The dramatic impact on RPEF and PenH is again consistent with diminished pulmonary function seen during infection with other microbes, including SARS-CoV and influenza virus (23, 29). While infection impacted pulmonary function, none of the treatments improved any of the parameters tested.

While we did not observe statistically significant differences in lung function parameters in treatment groups, we did observe a less dramatic shift for each parameter in the Fp T = −3 group between 24 hpi and 48 hpi, which represents the shift into the proinflammatory disease phase. To measure this transition for each parameter, we quantified the ratio of the value recorded at 48 hpi (entry into the proinflammatory state) relative to the value at 24 hpi (preinflammatory state; see Table S1 in the supplemental material). For untreated mice, there was a significant change in PenH, F, and RPEF between the 24- and 48-h time points that was not evident in the uninfected group of mice. The change in each parameter was reduced for the Fp T = −3 + Sm 48 group compared to all other groups, indicating a less dramatic shift into the proinflammatory phase of disease. In summary, we were able to demonstrate impaired pulmonary function upon intranasal infection of mice with Y. pestis. While none of the treatment regimens significantly impacted these parameters overall, pretreatment of mice with Fp dampened the shift into a proinflammatory state of disease.

## DISCUSSION

Pneumonic plague is primarily a host-driven disease that can be difficult to treat effectively. It is generally accepted that delayed antibiotic delivery (>24 h after the onset of symptoms in humans) results in poor survival outcomes (9, 30). Using the corticosteroid fluticasone propionate (Fp) in a murine intranasal infection model, we showed that pretreatment of mice with intranasal Fp in conjunction with delayed antibiotic treatment significantly enhanced survival compared antibiotic alone. We further showed that Fp pretreatment and delayed Sm delivery resulted in decreased bronchoalveolar lavage fluid (BALF) concentrations of IL-6 compared to untreated mice and decreased neutrophil frequency in the BALF. Taken together, these data indicate that host inflammatory responses complicate treatment of late-stage pneumonic plague.

During severe pneumonia, overactivation of immune response can lead to pulmonary damage that compromises pulmonary function, resulting in ARDS and/or death. For instance, increased levels of inflammatory cytokines and chemokines (such as TNF-α, IL-6, and IL-8) and extensive neutrophil infiltration into the airways correlate with poor clinical outcomes (31). Pneumonic plague, a severe and rapidly progressing pneumonia, is likewise difficult to treat during late-stage disease, due to its unique disease progression. Pneumonic plague has been modeled effectively in murine intranasal and aerosol infection platforms (19, 20, 32, 33). Shortly after deposition in the lower airways of infected mice, there is a slight decline or stalling of bacterial burdens in the lung within the first 24 hpi (32), followed by a nearly exponential increase in bacterial burdens that plateaus between 48 and 60 hpi (20, 32). This rapid replication is due to early circumvention of host inflammatory responses, attributed in large part to the Y. pestis type 3 secretion system (T3SS), and occurs in the absence of symptoms or notable inflammatory responses (20, 33). At between 24 and 36 hpi, bacteria can be found in the blood and spleen, where Y. pestis undergoes exponential bacterial growth (20, 32) that coincides with the onset of a proinflammatory disease phase defined by the massive upregulation of proinflammatory cytokines (20, 33). The proinflammatory phase progresses in severity and results in uniform mortality in infected mice by roughly 72 hpi. Due to its rapid progression and extremely high bacterial and blood burdens, there is a time frame of around 42 to 48 hpi where antibiotic treatment loses efficacy (13, 32). We sought to determine if pulmonary damage caused by host inflammatory responses also contributed to the limited antibiotic efficacy and short time window for effective treatment seen during the proinflammatory phase of disease.

Steroid treatment has been shown to be beneficial for treating inflammatory conditions resulting from severe diseases, including bacterial meningitis (34, 35), and clinically to treat severe lung injury caused by Mycoplasma pneumoniae (36–38). The COVID-19 pandemic has emphasized the potential of corticosteroid use in severe pneumonia. Several clinical studies have been conducted concerning the use of intravenous corticosteroids, such as dexamethasone (39–42), methylprednisolone (40–44), and hydrocortisone (42, 45), as adjunctive COVID-19 treatment. In some cases, these treatments have been shown to reduce COVID-19 symptoms and patient mortality, but variabilities between studies have made making decisive conclusions regarding corticosteroid use difficult (42, 46–49). We were able to rescue 30% of mice by using pretreatment with Fp in mice receiving antibiotic therapy initiated during late-stage infection. Quantification of bacterial burdens in the lung revealed that all treatment groups showed decreased levels of bacteria by 60 hpi, with groups receiving Fp plus Sm showing the most dramatic effect. The ability to improve survival of a particular treatment group afforded us the opportunity to identify host correlates of survival during treatment. Specifically, we show that Fp T = −3 groups demonstrated reduced levels of neutrophils as a percentage of cells in the BALF and reduced levels of BALF IL-6. IL-6 is highly induced in clinical cases of pneumonic plague (50) and is abundant in serum of severe pneumonia patients. IL-6 is known to be a critical mediator of inflammation (51) that influences neutrophil survival and recruitment (52–54). The function of IL-6 during the later stages remains undefined, and our data indicate that reduced IL-6 expression correlates with survival. It is notable that while reduced BALF neutrophils and IL-6 levels correlated with survival, depletion/ablation of either in conjunction with delayed antibiotic delivery did not rescue mice to the levels seen with Fp pretreament. This may indicate that though both may contribute to later disease pathology, they are necessary at earlier time points for combating disease. This may also indicate that both of these parameters are markers/correlates of survivability, but are not necessarily responsible for facilitating survival. It is likely that pulmonary conditions that support survival are multifactorial, and therefore more work is necessary to delineate precisely which combination of factors is responsible for improving antibiotic efficacy. It is also likely that the effect of glucocorticoid delivery on the behavior of specific cell types may impact survival, as glucocorticoid treatment is known to impact a variety of cellular responses in neutrophils, macrophages, and other cell types (55–57).

Murine survival outcomes may be improved by investigating the timing of antibiotic and corticosteroid delivery, dosage of corticosteroids, and different routes of drug delivery (such as aerosolization of antibiotics or parenteral delivery of corticosteroids). We chose streptomycin for our treatment model due to its long history as a “gold standard” for treatment of plague and its demonstrated efficacy in a murine model of pneumonic plague (9, 13, 58). While streptomycin is still considered one of the first-line treatments against pneumonic plague, other first-line agents, including gentamicin, ciprofloxacin, and doxycycline, are more commonly used today (59). Consideration of the specific antibiotic used is important, as other antibiotics and antibiotic classes may yield different results and may be more relevant to current treatment scenarios. For example, levofloxacin and moxifloxacin are now considered particularly good first-line options in the event of pneumonic plague or other community-acquired pneumonias due to their robust lung penetration and activity against a number of atypical pathogens (59). Also of note, streptomycin is an aminoglycoside protein synthesis inhibitor that will impact expression of the *Yersinia* outer proteins (Yops), which are critical *Yersinia* virulence determinants. While this should be advantageous to treatment, it is conceivable that loss of the anti-inflammatory Yops tips the balance of the pulmonary compartment to a slightly more inflammatory state, and in this case, other classes of antibiotic may be better suited for use in tandem with corticosteroids. Thus, it will be important to evaluate and compare between different antibiotic classes when considering anti-inflammatory treatment in conjunction with delayed antibiotic delivery.

To understand the role of pulmonary damage in the progression of pneumonic plague, we used whole-body plethysmography to monitor parameters of pulmonary function throughout the time course of infection and among treatment groups. Infection with Y. pestis dramatically impacted nearly all of the metrics we examined, indicative of its detrimental impact on pulmonary function. Many of the patterns we observed, including increased PenH coupled with decreased RPEF, were also found upon infection with SARS-CoV, influenza virus A, and SARS-CoV2 infection (23, 60) and are indicative of compromised respiration consistent with restrictive and obstructive airway disease patterns (23). Though none of the treatments tested significantly impacted pulmonary function compared to untreated, infected mice, pretreatment with fluticasone resulted in a less dramatic transition into the proinflammatory disease phase overall.

In summary, we showed that pretreatment of mice with intranasal fluticasone propionate increased survival of antibiotics delivered beyond the window of typical efficacy. These results indicate that host inflammatory responses complicate treatment of late-stage pneumonic plague, suggesting that coupling anti-inflammatory treatment with aggressive antibiotic therapy may improve survival outcomes of pneumonic plague. Little research has been performed to identify adjunctive therapies to overcome the host response during Y. pestis infection (61–64), and research is warranted to identify and evaluate additional supportive therapies in clinical cases. Further, these results may be relevant to other types of pneumonia for which host inflammatory responses complicate treatment of late and/or severe disease.

## MATERIALS AND METHODS

### Bacterial strains.

All work using fully virulent strains of Yersinia pestis was performed in compliance with NIAID and federal select agent policies and was performed in the UAMS biosafety level 3 (BSL3) laboratory registered with and approved by the CDC. The work adhered to the Yersinia pestis standard operating procedure, BSL3 safety manual, and select agent biosecurity plan approved by the BSL3 subcommittee of the UAMS institutional biosafety committee. The Colorado 92 (CO92) strain of Y. pestis was obtained from the lab of William Goldman (UNC—Chapel Hill). Y. pestis strains were grown on brain heart infusion (BHI) agar (Difco) at 26°C for 2 to 3 days. For infection, Y. pestis CO92 was inoculated in 10 mL BHI broth for 12 to 16 h supplemented with 2.5 mM CaCl_2_ and incubated at 37°C with constant shaking. Infections were performed in the UAMS BSL3 facility.

### Animals and animal infections.

All animal experiments were conducted with approval from the UAMS Institutional Animal Ethics Committee. Four- to 6-week-old C57BL/6J and IL-6 knockout (B6.129S2-*Il6^tm1Kopf^*/J) female mice were obtained from Jackson Laboratories. Mice were provided with food and water and maintained at 25 to 26°C with 40 to 70% humidity. To prepare mice for infection and fluticasone propionate treatment, mice were anesthetized using ketamine-xylazine injected via the intraperitoneal (i.p.) route. Once anesthetized, mice were inoculated via the intranasal route with lethal doses (10^4^ CFU) of Y. pestis delivered in 20 μL phosphate-buffered saline (PBS). For fluticasone propionate treatment, mice were given 200 μg fluticasone propionate (Tocris) dissolved in 4 μL DMSO via the intranasal route once per day after anesthesia. Streptomycin sulfate (G Biosciences) was delivered via the i.p. route (53.3 mg/kg of body weight per dose) every 8 h beginning at 24 and 48 hpi as needed per treatment group for 15 total doses. For analysis of bacterial burdens and host parameters, mice were euthanized via i.p. injection of 150 mg/kg sodium pentobarbital. To determine bacterial burdens in lungs of mice, lungs were homogenized in 1 mL PBS at 24, 48, and 60 h postinfection. Organ homogenates were serially diluted and plated on BHI agar containing 100 μg/mL cycloheximide (Acros) to determine CFU. For neutrophil depletion, 20 μg Ly-6G antibody (clone 1A8; BioLegend) was injected via the intravenous route as described previously (65, 66).

### CBA and ELISAs.

Th1/Th2/Th17 and mouse inflammatory cytokine bead array (CBA) kits were obtained from BD Biosciences. BALF supernatants were assayed per the manufacturer’s directions, and samples were analyzed on BD LSRFortessa and FACS Diva for data collection. Analysis was performed using BD FCAP Array software (version 3.0) to determine cytokine concentrations. MIP-1α and MIP-1β enzyme-linked immunosorbent assay (ELISA) kits (Invitrogen) were obtained, and BALF supernatants were assayed per the manufacturer’s directions. Samples were analyzed using a FLUOStar Omega plate reader (BMG Labtech) to determine cytokine concentrations.

### Preparation of BALF and whole lungs for flow cytometry.

Bronchoalveolar lavage fluid (BALF) was retrieved from mice postmortem by catheterization of the trachea as described previously (67). BALF was centrifuged for 5 min at 500 × *g* to pellet cells. Whole-lung cell suspensions were obtained from lungs retrieved from mice postmortem. Each lung was digested in 1 mL of a solution containing 1.5 mg/mL collagenase A (Gibco), 0.4 mg/ml DNase I (Alfa Aesar), 5% fetal bovine serum (FBS) (Corning), and 10 mM HEPES (Gibco), with the remaining volume made up with Hanks balanced salt solution (HBSS) (Gibco) with gentle shaking in an incubator at 37°C with 5% CO_2_ for 1 h. After digestion, cell suspensions were strained through a 70-μm cell strainer and pelleted at 500 × *g*. Supernatants were removed, and the cell pellet was treated with 1× red blood cell (RBC) lysis buffer for 5 min to lyse red blood cells. After RBC lysis inactivation, lung cells were pelleted and processed. After cell isolation, cells were resuspended in 3% FBS in PBS containing the following antibodies (1:500 dilution) for 30 min at 4°C to stain cell surface markers: CD45-phycoerthrin (clone 30-F-11; BD Biosciences), CD3-allophycocyanin-Cy7 (clone 17A2; BD Biosciences), CD11b-Alexa Fluor 700 (clone M1/70; BD Biosciences), CD11c-brilliant violet 786 (clone HL3; BD Biosciences), F4/80-allophycocyanin (clone BM8; Invitrogen), and Ly-6G–phycoerythrin–Cy7 (clone 1A8; BD Biosciences). After antibody staining, cells were centrifuged at 500 × *g* for 5 min, and the supernatant was removed. Cells were subsequently stained using LIVE/DEAD Fixable Aqua dead cell stain kit (Invitrogen) as per the manufacturer’s directions. Cells were pelleted at 500 × *g* for 5 min and resuspended in 2% formalin in PBS for fixation for 20 min at room temperature. After fixation, cells were pelleted and resuspended in 200 μl 3% FBS in PBS for flow cytometry using BD LSRFortessa and FACS Diva for data collection. Flow cytometry data were further analyzed using FlowJo software version 10.7 (BD Biosciences). The following cell populations were identified as described previously (15): T cells (CD45^+^, CD3^+^), alveolar macrophages (F4/80^+^ CD11b^mid/low^ CD11c^high^), CD11b^high^ interstitial/exudate macrophages (F4/80^+^ CD11b^high^ CD11c^low/mid^), F4/80^+^ CD11b^high^ CD11c^high^ macrophages, monocytes (F4/80^−^ CD11b^high^ CD11c^low^ Ly-6G^−^), CD11b^high^ and CD11b^low^ dendritic cells (F4/80^−^ CD11c^high^ CD11b^high or low^), and neutrophils (F4/80^−^ CD11c^low^ CD11b^high^ Ly-6G^+^).

### Lung pathology.

After sacrifice, mouse lungs were inflated with 10% formalin and harvested at 48 and 60 hpi. Lungs were placed in 5 mL 10% formalin and submitted to the UAMS Experimental Pathology Core for paraffin embedding, tissue slicing and mounting, and hematoxylin and eosin staining. Three sections per lung (each section cut 100 μm apart) were obtained. Lung lesions were counted, and the area of inflammation was determined using the Fiji/Image J (68) Magic Wand tool.

### Whole-body plethysmography.

Mice were acclimated in whole-body plethysmographer (Fine Pointe; DSI) chambers 5 days prior to taking plethysmography readings for 10 min. At data collection time points, chambers were calibrated prior to data collection. Respiratory parameters were measured during quiet wakefulness for 10 min at each time point. The index of constriction (PenH), inspiratory time (Ti), respiratory rate (F), estimated peak inspiratory flow (PIFb), RPEF, tidal volume (TVb), expiratory time (Te), end inspiratory pause (EIP), and flow at 50% expired breath volume (EF50) were derived from box flow signal.

### Statistical analyses.

Statistical analyses were done using one-way and two-way analysis of variance (ANOVA) and the log rank test where applicable. *P* values are represented by asterisks as follows: *, *P* < 0.05; **, *P* < 0.005; ***, *P* < 0.0005; ****, *P* < 0.0001. All statistical analyses were performed using GraphPad Prism v8.4.3 software.
