# Supplementary material for: Treatment with Fluticasone Propionate Increases Antibiotic Efficacy during Treatment of Late-Stage Primary Pneumonic Plague
Source: Antimicrob Agents Chemother. 2022 Jan 18;66(1):e01275-21. doi: 10.1128/AAC.01275-21 (PMC8765263; doi:10.1128/AAC.01275-21)
Supplement: Supplemental file 1 — Supplemental table and figures. Download AAC.01275-21-s0001.pdf, PDF file, 5.9 MB [file aac.01275-21-s0001.pdf]

Table S1: Change in Lung Function Parameters Between Preinflammatory and Proinflammatory Stages of Pneumonic Plague

	PenH			F			RPEF		
	Average Value (24 hpi)	Average Value (48 hpi)	ΔPenH (48/24 hpi)	Average Value (24 hpi)	Average Value (48 hpi)	ΔF (48/24 hpi)	Average Value (24 hpi)	Average Value (48 hpi)	-1 ÷ΔRPEF (48/24 hpi)
**Untreated**	0.8073	7.85	9.723	470.2	282.2	-188	0.3627	0.1576	-2.301
**Fp T=-3 + Sm 48**	1.971	5.292	2.684	379.5	286.2	-93.3	0.2887	0.2071	-1.394
**Sm 48**	0.7512	4.68	6.230	449.3	324	-125.3	0.3752	0.2274	-1.649
**Fp + Sm 48**	0.7101	7.234	10.18	454.4	279	-175.4	0.3821	0.1568	-2.436

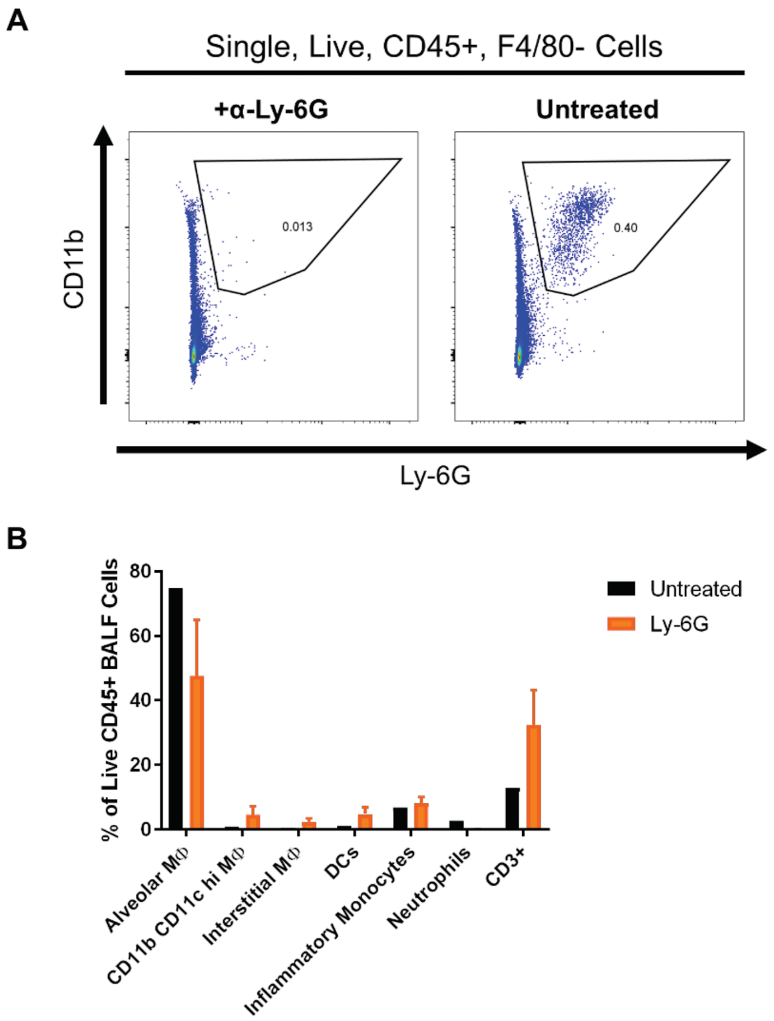

Supplemental Figure 1. Depletion of neutrophils using α-Ly-6G depleting antibody. Whole lungs were retrieved from uninfected C57BL/6 mice receiving α-Ly-6G depleting antibody one day prior and at day of infection (0 hpi) and digested using a solution containing collagenase, DNase I, FBS, HEPES, and HBSS. A) Representative flow plots from untreated and α-Ly-6G treated mice at day of infection are pre-gated on single, live, CD45+, F4/80- cells. Gate represents neutrophil population. B) Frequency of leukocytes in untreated and α-Ly-6G treated mice after whole lung digestion. Error bars represent SD. Data are representative of two experiments.

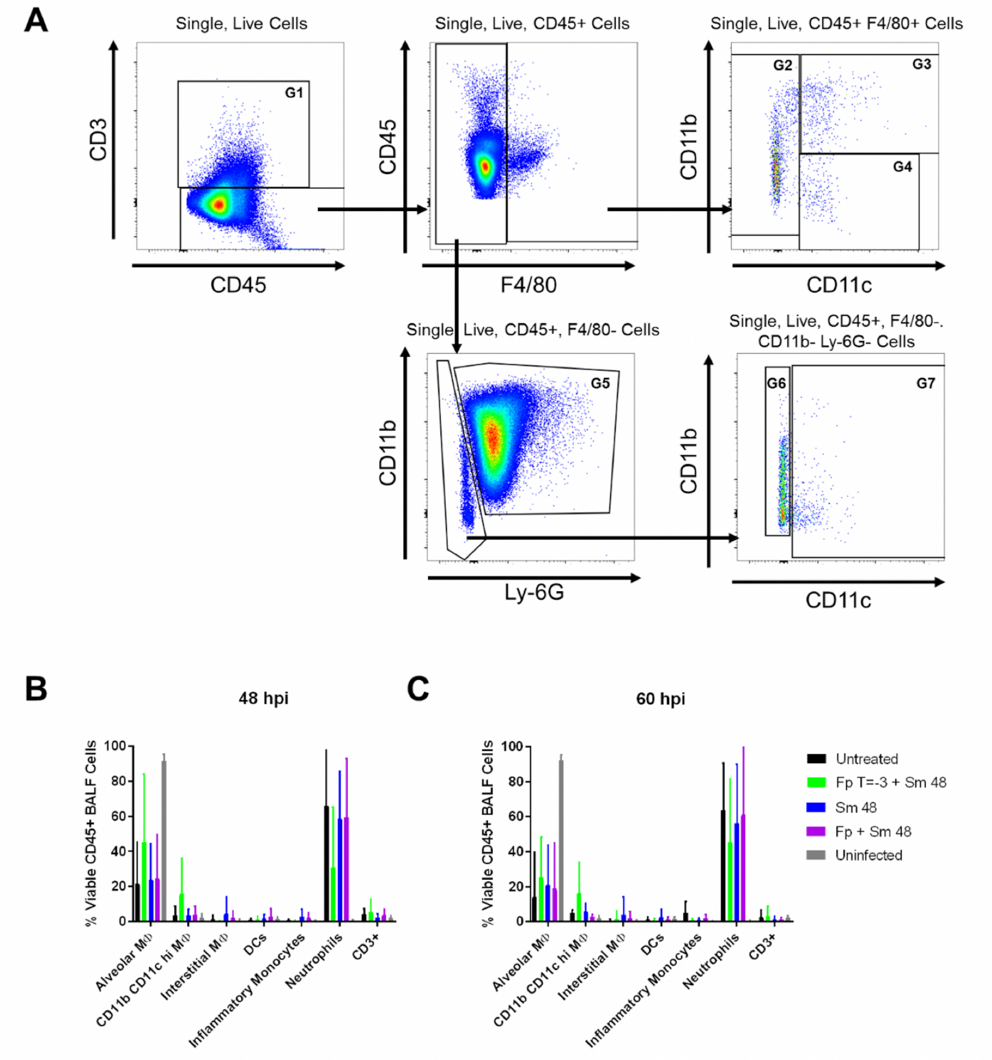

Supplemental Figure 2. Gating strategy for BALF lymphocytes and BALF cell population profiles at 48 and 60 hpi. A) Gating strategy for BALF immune cell frequencies from infected mouse (Fp + Sm 48 cohort) at 48 hpi. Cells were pre-gated on live, single cells. Gates of interest are labeled in panels. G1=CD3+ cells. G2=alveolar macrophages. G3=CD11b+ CD11c+ macrophages. G4=interstitial macrophages. G5=neutrophils. G6=inflammatory monocytes. G7=dendritic cells (DCs). Lymphocyte population frequencies in BALF at B) 48 hpi and C) 60 hpi. Significant populations (alveolar macrophages and neutrophils) are shown in Figure 6 for ease of viewing.
